# Telomere length and the risk of cardiovascular diseases: A Mendelian randomization study

**DOI:** 10.3389/fcvm.2022.1012615

**Published:** 2022-10-24

**Authors:** Yingjian Deng, Qiang Li, Faguang Zhou, Guiyang Li, Jianghai Liu, Jialan Lv, Linlin Li, Dong Chang

**Affiliations:** Department of Cardiology, Xiamen Cardiovascular Hospital, School of Medicine, Xiamen University, Xiamen, China

**Keywords:** telomere length, cardiovascular diseases, genetic variants, Mendelian randomization, causal association

## Abstract

**Background:**

The causal direction and magnitude of the associations between telomere length (TL) and cardiovascular diseases (CVDs) remain uncertain due to susceptibility of reverse causation and confounding. This study aimed to investigate the associations between TL and CVDs using Mendelian randomization (MR).

**Materials and methods:**

In this two-sample MR study, we identified 154 independent TL-associated genetic variants from a genome-wide association study (GWAS) consisting of 472,174 individuals (aged 40–69) in the UK Biobank. Summary level data of CVDs were obtained from different GWASs datasets. Methods of inverse variance weighted (IVW), Mendelian Randomization-Egger (MR-Egger), Mendelian Randomization robust adjusted profile score (MR-RAPS), maximum likelihood estimation, weighted mode, penalized weighted mode methods, and Mendelian randomization pleiotropy residual sum and outlier test (MR-PRESSO) were conducted to investigate the associations between TL and CVDs.

**Results:**

Our findings indicated that longer TL was significantly associated with decreased risk of coronary atherosclerosis [odds ratio (OR), 0.85; 95% confidence interval (CI), 0.75–0.95; *P* = 4.36E-03], myocardial infarction (OR, 0.72; 95% CI, 0.63–0.83; *P* = 2.31E-06), ischemic heart disease (OR, 0.87; 95% CI, 0.78–0.97; *P* = 1.01E-02), stroke (OR, 0.87; 95% CI, 0.79–0.95; *P* = 1.60E-03), but an increased risk of hypertension (OR, 1.12; 95% CI, 1.02–1.23; *P* = 2.00E-02). However, there was no significant association between TL and heart failure (OR, 0.94; 95% CI, 0.87–1.01; *P* = 1.10E-01), atrial fibrillation (OR, 1.01; 95% CI, 0.93–1.11; *P* = 7.50E-01), or cardiac death (OR, 0.95; 95% CI, 0.82–1.10; *P* = 4.80E-01). Both raw and outlier corrected estimates from MR-PRESSO were consistent with those of IVW results. The sensitivity analyses showed no evidence of pleiotropy (MR-Egger intercept, *P* > 0.05), while Cochran’s *Q* test and MR-Egger suggested different degrees of heterogeneity.

**Conclusion:**

Our MR study suggested that longer telomeres were associated with decreased risk of several CVDs, including coronary atherosclerosis, myocardial infarction, ischemic heart disease, and stroke, as well as an increased risk of hypertension. Future studies are still warranted to validate the results and investigate the mechanisms underlying these associations.

## Introduction

Telomeres are DNA-protein complexes composed of deoxyribonucleic acid repeats and are located at the termini of linear chromosomes. Telomeres protect the genes from damage and are thus important for chromosomal stability and cellular integrity (1). Telomere length (TL) has long been regarded as a reliable biomarker for cellular aging due to its progressive shortening at each cell cycle division (2–4). At a population level, TL is often measured in blood leukocytes, which has been suggested highly correlated with TL across different tissues (5). Previous studies have shown that TL is largely genetically determined (6, 7). The shortening of telomeres can induce DNA damage and provoke apoptosis and cell senescence, which may contribute to many aging-associated diseases (8, 9). Epidemiological studies have shown that telomere shortening is related to an increased risk of diabetes (10), cancer (11), and non-vascular, non-neoplastic causes of mortality (12).

Cardiovascular diseases (CVDs) are defined as disorders of the heart and blood vessels that include hypertension, coronary heart disease, myocardial infarction, ischemic heart disease, atrial fibrillation, heart failure, and stroke (13). With the aging of the global population, the socioeconomic burden brought about by CVDs continues to increase every year. Globally, CVDs are continuing to be the leading cause of death in 2019, causing an estimated 17.9 million deaths each year (13). Telomeres have been suggested to play an important role in the development and prognosis of CVDs, which has drawn considerable research interest in recent years (14–17). A multicenter, community-based cohort study from the UK Biobank showed that reduced leukocyte TL was associated with increased cardiovascular mortality (18). Meanwhile, a meta-analysis involving 43,725 individuals indicated a negative correlation between TL and coronary heart disease, but no significant association with cerebrovascular disease was found (19). However, a recent study suggested that TL was not associated with cardiovascular mortality (20). Therefore, it still remains unclear whether TL is causally involved in the development of CVDs, as the results have been varied and inconsistent across observational studies mainly due to confounding, and reverse causation. A clear understanding of the relationship between TL and the risk of CVDs is essential to the prevention and treatment of CVDs among the elderly population.

Mendelian randomization (MR) is a robust method of establishing a causal association between an exposure and an outcome using genetic variants related to the exposure of interest (21). These genetic variants are randomly inherited from parents and are thus not affected by confounders of the exposure-outcome association. Compared to traditional epidemiological approaches, MR is better at establishing causal relationships between the exposure and outcome variables with much less confounding and biases and has gained increasing popularity in research. In recent years, MR study has been widely used to assess causality in epidemiologic settings. In the present study, we conducted the largest two-sample MR study to assess the possible associations between TL and the risk of CVDs using the latest genome-wide association study (GWAS) summary data.

## Materials and methods

### Mendelian randomization estimates

**FIGURE 1:**
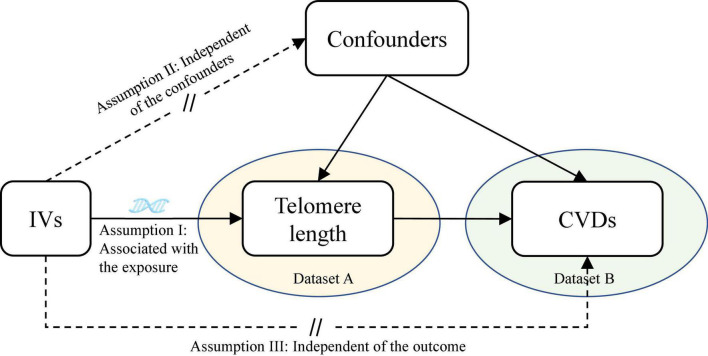
Assumptions of the Mendelian randomization (MR) analysis for TL and the risk of CVDs. The MR study assumes that genetic variants are associated with only TL and not with confounders or alternative causal pathways, that is, the IVs affect the risk of CVDs only directly through TL. TL, telomere length; CVDs, cardiovascular diseases; IVs, instrument variables.

This is a two-sample MR study design based on the data from different large-scale GWAS datasets. The study design overview of the MR analysis is presented in Figure 1. The potential genetic variants selected to estimate the effects must comply with three key assumptions (22): (1) the genetic variants should be associated with TL (*P* < 5 × 10^–8^); (2) the genetic variants must not be associated with the outcome and confounding factors of the exposure-outcome association; (3) the genetic variants must be associated with CVDs only through TL. All contributing studies have received ethical approval from their respective medical ethical committees and obtained informed consent from all study participants.

### Genetic instrument selection

Genetic variants associated with TL were retrieved from a hitherto largest GWAS (Dataset ID: ieu-b-4879) including 472,174 individuals (aged 40–69) in the UK Biobank, with a similar proportion of males (45.8%) and females (54.2%) (23). The self-reported ethnicity was predominantly White European (94.3% White, 1.9% Asian, 1.5% Black, 0.3% Chinese, 0.6% Mixed, and 0.9% other ethnicities) (23). Age, sex, and ethnic group were adjusted in this study. In the UK Biobank cohort, DNA was extracted from the peripheral blood leukocytes, and TL was measured as the T/S ratio using the quantitative polymerase chain reaction methodology (24). We selected only independent (*R*^2^ < 0.001) and genome-wide significant (*P* < 5 × 10^–8^) single nucleotide polymorphisms (SNPs) from this GWAS as the instrumental variables (IVs) for TL, which resulted in 154 SNPs (Supplementary Table 1). The strength of each genetic instrument was measured using F-statistics (F = *R*^2^/(1-*R*^2^) × [(N-K-1)/K], where R^2^ is the proportion of the exposure explained by the genetic instrument, N is the sample size, and K is the number of SNPs) (25). The PhenoScanner database V2 was used to assess and remove those SNPs that are associated with other phenotypes, including the potential confounders and mediators (i.e., diabetes, hyperlipidemia, hypertension, smoking, and body mass index) (26, 27). We also removed SNPs for palindromic or incompatible alleles when harmonizing TL and outcomes. In the present study, 130, 128, 128, 128, 119, 116, 130, and 138 SNPs were eventually obtained as the IVs for TL to assess the associations between TL and hypertension, myocardial infarction, coronary atherosclerosis, ischemic heart disease, heart failure, stroke, atrial fibrillation, and cardiac death, respectively.

### Outcome data

Summary-level data for CVDs were obtained from recently published GWAS datasets. The GWAS summary data of hypertension (55,917 cases and 162,837 controls), coronary atherosclerosis (23,363 cases and 187,840 controls), ischemic heart disease (31,640 cases and 187,152 controls), myocardial infarction (11,622 cases and 187,840 controls), and cardiac death (7,563 cases and 211,229 controls) were from the FinnGen consortium. The heart failure GWAS dataset (47,309 cases and 930,014 controls) was from the Heart Failure Molecular Epidemiology for Therapeutic Targets (HERMES) consortium. The atrial fibrillation (60,620 cases and 970,216 controls) data was from a GWAS meta-analysis of The Nord-Trøndelag Health Study (HUNT), the Michigan Genomics Initiative (MGI), deCODE, UK Biobank, DiscovEHR, and the AFGen Consortium (28). The GWAS data of stroke (40,585 cases and 406,111 controls) was also from different consortiums (MEGASTROKE, etc.) (29). The participants included in this study were mostly of European ancestry. All datasets included in the current study are summarized in Table 1.

**TABLE 1:** Details of studies included in Mendelian randomization (MR) analyses.

Phenotype	Consortium of study	Sample size	Ethnicity	Year
Telomere length	UKB	472,174	European (mostly)	2021
Hypertension	FG	218,754	European	2021
Myocardial infarction	FG	199,462	European	2021
Coronary atherosclerosis	FG	211,203	European	2021
Ischemic heart disease	FG	218,792	European	2021
Heart failure	HERMES	977,323	European	2020
Atrial fibrillation	AF Gen, etc.	1,030,836	European (mostly)	2018
Stroke	MEGASTROKE, etc.	446,696	European (mostly)	2018
Cardiac death	FG	218,792	European	2021

UKB, UK biobank; FG, FinnGen; HERMES, heart failure molecular epidemiology for therapeutic targets; AF Gen, atrial fibrillation genetics.

### Statistical analysis

The IVW method was used as the principal MR analytic approach to assess the associations between TL and the risk of CVDs. All statistical analyses were performed using R software (version 4.1.2). Analyses were performed using TwoSampleMR R package (30), which included inverse variance weighted (IVW), Mendelian Randomization-Egger (MR-Egger), and weighted mode. A *P*-value less than 0.05 (two-sided) was considered statistically significant. We used the IVW (Q) and MR-Egger methods to test for heterogeneity, the MR-Egger regression (MR-Egger intercept test) to evaluate the horizontal pleiotropy (statistical significance was set at a level of *P* < 0.05), and the leave-one-out analysis to exclude the possible influence of individual SNPs on the overall results. For sensitivity analyses, *P* < 0.05 in Cochran’s *Q* test suggested significant heterogeneity. The MR-IVW fixed-effects model was adopted for SNPs without heterogeneity (*P* > 0.05 in Cochran’s *Q* test), while the MR-IVW random-effects model was used for heterogeneous SNPs. We further performed the robust adjusted profile score (MR-RAPS), maximum likelihood estimation, and penalized weighted median (PWM) method to assess the effects of TL on the risk of CVDs (31–33). Additionally, we used the Mendelian randomization pleiotropy residual sum and outlier (MR-PRESSO) test to identify and remove the horizontal pleiotropic outliers (34).

## Results

In our MR study, 154 independent leukocyte TL-associated SNPs were obtained as IVs (Supplementary Table 1). The F-statistics of all these genetic variants were above the threshold of 10 (range: 29.86–1628.82), indicating that our IVs were strongly predictive of TL. The SNPs that were associated with confounding factors and SNPs for palindromic or incompatible alleles were removed.

**FIGURE 2:**
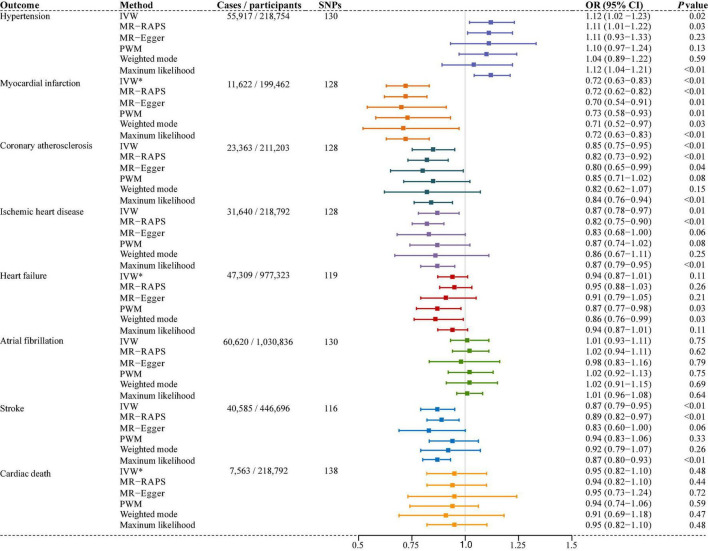
Associations of genetically predicted telomere length (TL) and the risk of cardiovascular diseases (CVDs). SNPs, single nucleotide polymorphisms; IVW, inverse variance weighted (random-effects model); IVW*, inverse variance weighted (fixed-effects model); MR-RAPS, Mendelian randomization robust adjusted profile score; MR-Egger, Mendelian randomization-Egger; PWM, penalized weighted median; OR, odds ratio; CI, confidence interval.

**FIGURE 3:**
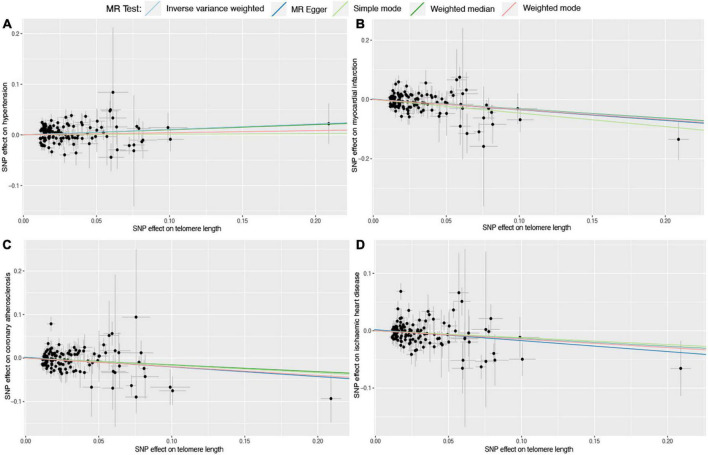
Scatter plots for Mendelian randomization (MR) analyses of the correlation between telomere length (TL) and cardiovascular diseases (CVDs). **(A)** TL-hypertension; **(B)** TL-myocardial infarction; **(C)** TL-coronary atherosclerosis; **(D)** TL-ischemic heart disease. The slope of each line corresponds to the estimated association effect between TL and the risk of CVDs of different MR methods.

**FIGURE 4:**
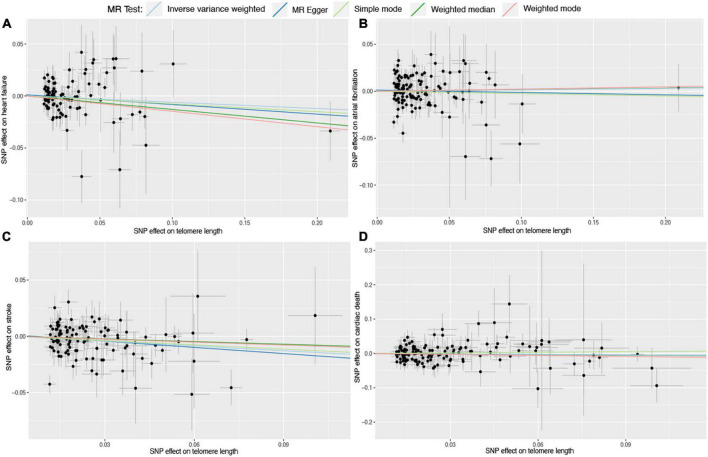
Scatter plots for Mendelian randomization (MR) analyses of the correlation between telomere length (TL) and cardiovascular diseases (CVDs). **(A)** TL-heart failure; **(B)** TL-atrial fibrillation; **(C)** TL-stroke; **(D)** TL-cardiac death. The slope of each line corresponds to the estimated association effect between TL and the risk of CVDs of different MR methods.

Six different methods of MR analyses, i.e., the IVW method, MR-RAPS, MR-Egger, PWM, weighted mode, and maximum likelihood, were implemented. The IVW method was used as the principal MR analytic approach. Using the IVW method, we found that genetically increased TL was significantly associated with lower odds of coronary atherosclerosis [IVW: odds ratio (OR), 0.85; 95% confidence interval (CI), 0.75–0.95; *P* = 4.36E-03], myocardial infarction (IVW: OR, 0.72; 95% CI, 0.63–0.83; *P* = 2.31E-06), ischemic heart disease (IVW: OR, 0.87; 95% CI, 0.78–0.97; *P* = 1.01E-02), and stroke (IVW: OR, 0.87; 95% CI, 0.79–0.95; *P* = 1.60E-03), but higher odds of hypertension (IVW: OR, 1.12; 95% CI, 1.02–1.23; *P* = 2.00E-02). However, we did not find enough evidence for the relationships between TL and heart failure (IVW: OR, 0.94; 95% CI, 0.87–1.01; *P* = 1.10E-01), atrial fibrillation (IVW: OR, 1.01; 95% CI, 0.93–1.11; *P* = 7.50E-01), and cardiac death (IVW: OR, 0.95; 95% CI, 0.82–1.10; *P* = 4.80E-01). The two-sample MR estimates for the associations between TL and the risk of CVDs were presented in Figure 2. The scatter plots for the MR TL-to-CVDs association were presented in Figures 3, 4.

The MR-PRESSO test identified one outlier SNP for ischemic heart disease (rs429358, Rssobs = 5.08E-03, *P* < 0.128), five outlier SNPs for atrial fibrillation (rs12369950, Rssobs = 2.05E-03, *P* < 0.13; rs2306646, Rssobs = 8,89E-04, *P* < 0.13; rs4743037, Rssobs = 1.06E-03, *P* < 0.13; rs6584579, Rssobs = 1.11E-03, *P* < 0.13; rs6751209, Rssobs = 7.48E-04, *P* < 0.13), and one outlier SNP for stroke (rs6584579, Rssobs = 1.69E-03, *P* < 0.116). No outlier SNPs were identified for hypertension, myocardial infarction, coronary atherosclerosis, heart failure, and cardiac death. Both raw and outlier corrected estimates from MR-PRESSO were consistent with the IVW results (Table 2). After removing the outlier SNPs, the IVW analysis showed basically the same results (Supplementary Table 2).

**TABLE 2:** The Mendelian randomization pleiotropy residual sum and outlier (MR-PRESSO) tests of the relationship between telomere length (TL) and cardiovascular diseases (CVDs).

Outcome	Raw estimates				Outlier corrected estimates			
	*N*	OR	95% CI	*P*-value	*N*	OR	95% CI	*P*-value
Hypertension	130	1.12	1.02–1.23	0.02	–	–	–	–
Myocardial infarction	128	0.73	0.63–0.84	<0.01	–	–	–	–
Coronary atherosclerosis	128	0.85	0.76–0.95	<0.01	–	–	–	–
Ischemic heart disease	128	0.87	0.78–0.96	<0.01	127	0.86	0.78–0.94	<0.01
Heart failure	119	0.95	0.88–1.03	0.21	–	–	–	–
Atrial fibrillation	130	1.03	0.94–1.12	0.55	125	1.02	0.95–1.10	0.58
Stroke	116	0.88	0.80–0.95	<0.01	115	0.89	0.82–0.96	<0.01
Cardiac death	138	0.96	0.85–1.10	0.56	–	–	–	–

OR, odds ratio; CI, confidence interval.

The IVW, MR-Egger, and MR-Egger intercept tests were performed as sensitivity analyses to detect the potential heterogeneity and horizontal pleiotropy (Table 3). According to the sensitivity analyses, the intercept test for MR-egger suggested no pleiotropy; however, Cochran’s *Q* test and MR-Egger test suggested different degrees of heterogeneity (Table 3). The leave-one-out sensitivity analysis was presented in Supplementary Figures 1–8, which validated the stability of the results.

**TABLE 3:** Heterogeneity and horizontal pleiotropy of the associations between telomere length (TL) and cardiovascular diseases (CVDs).

Outcome	Heterogeneity test (IVW)		Heterogeneity test (MR Egger)		MR Egger intercept test		
	*Q*-value	*P*	*Q*-value	*P*	I	SE	*P*
Hypertension	198.56	<0.01	198.56	<0.01	0.00	0.00	0.97
Myocardial infarction	141.33	0.11	141.21	0.10	0.00	0.00	0.75
Coronary atherosclerosis	150.69	0.04	150.25	0.04	0.00	0.00	0.56
Ischemic heart disease	163.00	0.01	162.45	0.01	0.00	0.00	0.53
Heart failure	121.03	0.07	121.37	0.07	0.00	0.00	0.60
Atrial fibrillation	268.73	<0.01	269.28	<0.01	0.00	0.00	0.62
Stroke	148.95	0.01	149.28	0.01	0.00	0.00	0.62
Cardiac death	103.03	0.87	103.28	0.88	0.00	0.00	0.62

IVW, inverse variance weighted; MR-RAPS, Mendelian randomization robust adjusted profile score; *Q*-value, the statistics of Cochrane’s *Q* test; I, intercept; SE, standard error.

## Discussion

In our study, we found that TL was associated with the risk of certain kinds of CVDs. To be specific, longer TL predicted a lower risk of coronary atherosclerosis, myocardial infarction, ischemic heart disease, and stroke, but a higher risk of hypertension. However, we did not find enough evidence to support the associations of TL with heart failure, atrial fibrillation, and cardiac death.

The findings that longer TL was associated with decreased risk of coronary atherosclerosis, ischemic heart disease, myocardial infarction, and stroke were consistent with previous studies. For instance, a recent meta-analysis based on 18 studies (involving 14,491 individuals) showed that the TL in patients with coronary artery disease was significantly shorter than that in the controls (standard mean difference = –0.45; 95% CI, –0.65–0.25) (35). A meta-analysis involving 32 studies (44,610 participants) reported that the shortest TL was related to a higher risk of myocardial infarction (risk ratio, 1.39; 95% CI, 1.16–1.67) (36). A cohort study with 29 years of follow-up reported that the lowest TL was modestly related to an increased risk of ischemic heart disease (hazard ratio, 1.55; 95% CI, 1.02–2.35). A meta-analysis based on 11 studies (25,340 individuals) indicated a significant association between shortened TL and stroke (OR: 1.5; 95% CI, 1.13–2.00) (37). All these findings supported the protective roles of increased TL in certain CVDs and suggest that special attention should be paid to those with shortened TL and certain measures to be taken to prevent the occurrence of CVDs in the future.

Of note, we found that longer TL was related to an increased risk of hypertension, which was opposite to the findings regarding coronary atherosclerosis, ischemic heart disease, myocardial infarction, and stroke. Previous studies have reached inconsistent conclusions on the correlation between TL and hypertension. For instance, some previous studies suggested that there was no association between TL and blood pressure (38–40). A previous meta-analysis of 10 studies indicated a significant negative correlation between TL and hypertension (41), which was also observed in recent studies (42–45). However, it has also been reported that longer TL is associated with higher blood pressure (46, 47). Meanwhile, several studies have found a non-linear correlation between TL and blood pressure (48–50). These findings suggest inconclusive and contradictory associations between TL length and hypertension, which warrant further research. Additionally, although our MR study did not find evidence to support the associations between TL and heart failure or cardiac death, the ORs for these associations were both less than 1.0, suggesting that TL might be a potential risk factor for heart failure and cardiac death. Previous studies have shown inconsistent results in the associations of TL with heart failure, atrial fibrillation, and cardiac death (14, 51–57), indicating the need for further research.

The mechanism of the associations between TL and CVDs remains elusive. Previous studies have reported that accelerated telomere shortening was associated with oxidative stress and chronic inflammation, both were critical factors that contribute to CVDs (58). Telomere shortening might also induce endothelial cell senescence and thereby promote human atherogenesis (59, 60). In addition, accelerated telomere shorting has been reported to be related to bone marrow-derived endothelial injury, which was important for the re-endothelialization of damaged vessels (61). It has also been suggested that TL was related to the development of type 2 diabetes, a common risk factor for CVDs (62). Cellular senescence in arteries induced by telomere dysfunction might contribute to the pathogenesis of hypertension (39). Besides, multiple social, environmental, and psychological factors could also affect these relationships. For example, it has been found that lack of physical activity, smoking, drinking, and high body mass index was negatively associated with TL, while regular exercise and a high-fiber diet were positively associated with the length of telomeres (63).

Several strengths of this MR study should be noted. First, we excluded the genetic variants that were related to potential confounders commonly found in epidemiological studies and selected only the SNPs strongly associated with TL. Second, the large sample size of our MR analysis enhanced our statistical power and provided reliable evidence of associations. Besides, we used the MR-PRESSO test to identify and remove variants that were horizontal pleiotropic outliers. Finally, we performed several sensitivity analyses, such as IVW, MR-Egger, and leave-one-out analysis, to confirm the robustness of these findings. Despite the strengths, there are some limitations to this study. First, our summary-level MR analysis assumed a linear relationship between TL and CVDs, which might not be the fact. Second, selection bias, such as the potential “healthy volunteer” bias and low recruitment rate, and bias from sample overlap could not be completely avoided. Third, different degrees of heterogeneity were observed in the MR-Egger and IVW (Q) methods, suggesting that our findings might be affected by pleiotropy. Furthermore, the participants in this study were mostly of European ancestry, which minimized the population stratification bias but limited the generalizability of our findings to other populations.

## Conclusion

Our MR study provided strong evidence of the relationships between TL shortening and increased risk of coronary atherosclerosis, myocardial infarction, ischemic heart disease, and stroke, as well as decreased risk of hypertension. We did not find the casual associations of TL with heart failure, atrial fibrillation, and cardiac death. Future studies are still warranted to validate the results and investigate the mechanisms underlying these associations.

## Data availability statement

The original contributions presented in this study are included in the article/Supplementary material, further inquiries can be directed to the corresponding author.

## Author contributions

YD and QL designed the study and analyzed and interpreted the data. YD drafted the manuscript. FZ, GL, JHL, JLL, and LL provided feedback on earlier drafts of the manuscript. DC reviewed and edited the manuscript. All authors contributed to the article and approved the submitted version.
