# Supplementary material for: Telomere length and the risk of cardiovascular diseases: A Mendelian randomization study
Source: Front Cardiovasc Med. 2022 Oct 24;9:1012615. doi: 10.3389/fcvm.2022.1012615 (PMC9637552; doi:10.3389/fcvm.2022.1012615)
Supplement: Supplementary file 2 [file Table_2.docx]

**Supplementary Table 2**. Associations of genetically predicted telomere length with ischemic heart disease, atrial fibrillation, and stroke after removing the outlier SNPs identified by the MR-PRESSO test.

| **Outcome** | **MR methods** | **Cases/participants** | **SNPs** | **OR** | **95% CI** | ***P* value** |
| --- | --- | --- | --- | --- | --- | --- |
| Ischemic heart disease | IVW | 31,640 / 218,792 | 127 | 0.86 | 0.78-0.95 | 2.50E-03 |
|  | MR-RAPS |  |  | 0.82 | 0.75-0.90 | 5.13E-05 |
|  | MR-Egger |  |  | 0.84 | 0.70-1.00 | 5.77E-02 |
|  | PWM |  |  | 0.87 | 0.74-1.01 | 7.09E-02 |
|  | Weighted mode |  |  | 0.87 | 0.68-1.12 | 2.77E-01 |
|  | Maxinum likelihood |  |  | 0.86 | 0.78-0.94 | 1.10E-03 |
| Atrial fibrillation | IVW | 60,620 / 1,030,836 | 125 | 1.03 | 0.95-1.11 | 4.91E-01 |
|  | MR-RAPS |  |  | 1.02 | 0.95-1.10 | 5.93E-01 |
|  | MR-Egger |  |  | 0.95 | 0.82-1.01 | 4.52E-01 |
|  | PWM |  |  | 1.02 | 0.92-1.12 | 7.54E-01 |
|  | Weighted mode |  |  | 1.03 | 0.90-1.16 | 6.98E-01 |
|  | Maxinum likelihood |  |  | 1.03 | 0.97-1.09 | 3.74E-01 |
| Stroke | IVW | 40,585 / 446,696 | 115 | 0.88 | 0.81-0.95 | 1.43E-03 |
|  | MR-RAPS |  |  | 0.89 | 0.82-0.97 | 5.55E-03 |
|  | MR-Egger |  |  | 0.80 | 0.67-0.94 | 9.43E-03 |
|  | PWM |  |  | 0.94 | 0.83-1.06 | 3.23E-01 |
|  | Weighted mode |  |  | 0.92 | 0.80-1.06 | 2.56E-01 |
|  | Maxinum likelihood |  |  | 0.87 | 0.81-0.95 | 6.68E-04 |

* MR-PRESSO, Mendelian randomization pleiotropy residual sum and outlier test; SNPs, single nucleotide polymorphisms; OR, odds ratio; CI, confidence interval.
