# Supplementary material for: Telomere length and the risk of cardiovascular diseases: A Mendelian randomization study
Source: Front Cardiovasc Med. 2022 Oct 24;9:1012615. doi: 10.3389/fcvm.2022.1012615 (PMC9637552; doi:10.3389/fcvm.2022.1012615)
Supplement: Supplementary file 4 [file Data_Sheet_2.PDF]

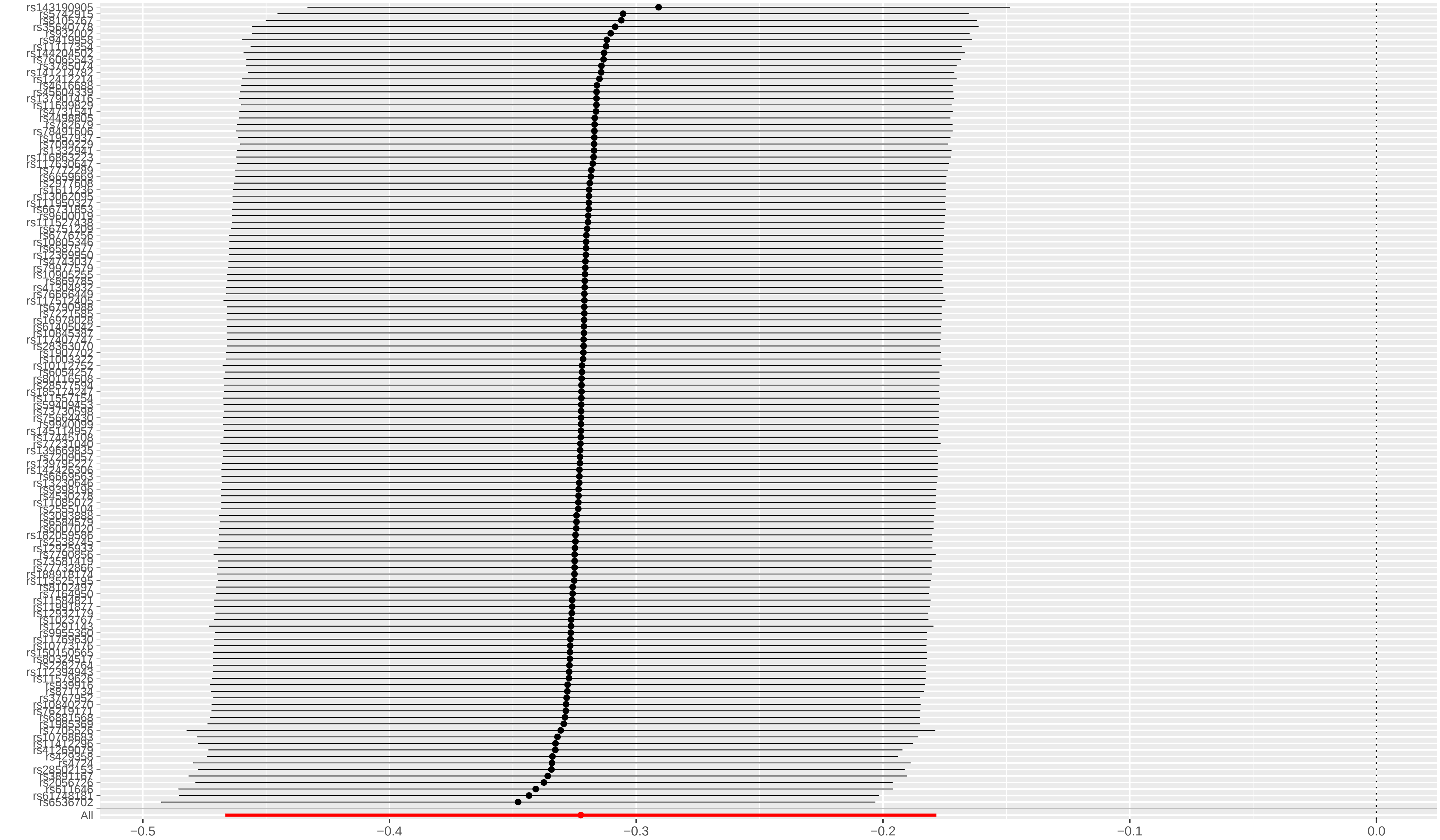

Supplementary Figure 2. MR leave−one−out sensitivity analysis for
'telomere length || id:ieu−b−4879' on 'Myocardial infarction, strict || id:finn−b−I9_MI_STRICT'
