# Supplementary figures and images for: Telomere length and the risk of cardiovascular diseases: A Mendelian randomization study
Source: Front Cardiovasc Med. 2022 Oct 24;9:1012615. doi: 10.3389/fcvm.2022.1012615 (PMC9637552; doi:10.3389/fcvm.2022.1012615)

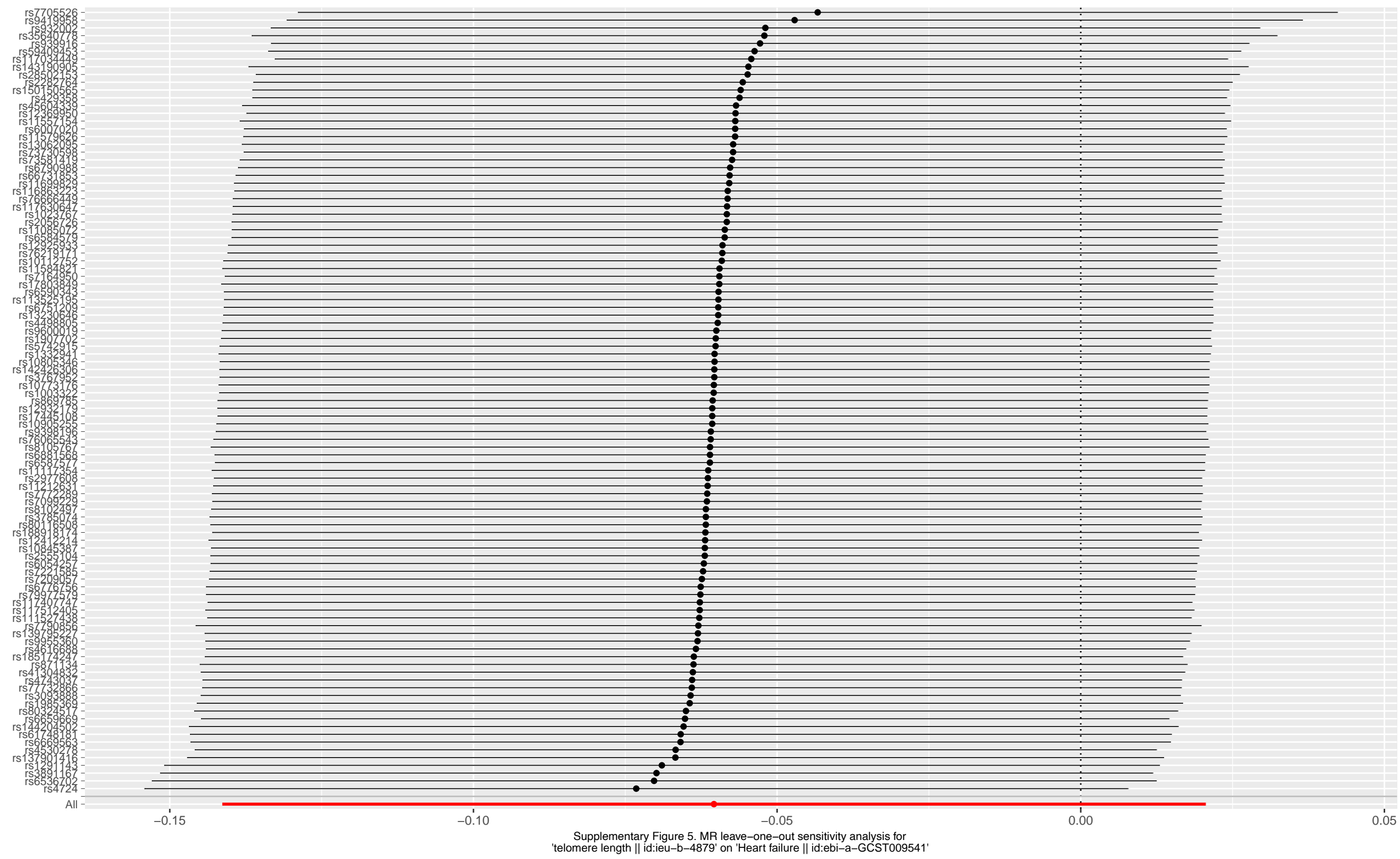

Supplement: Supplementary file 7 [file Data_Sheet_5.PDF]

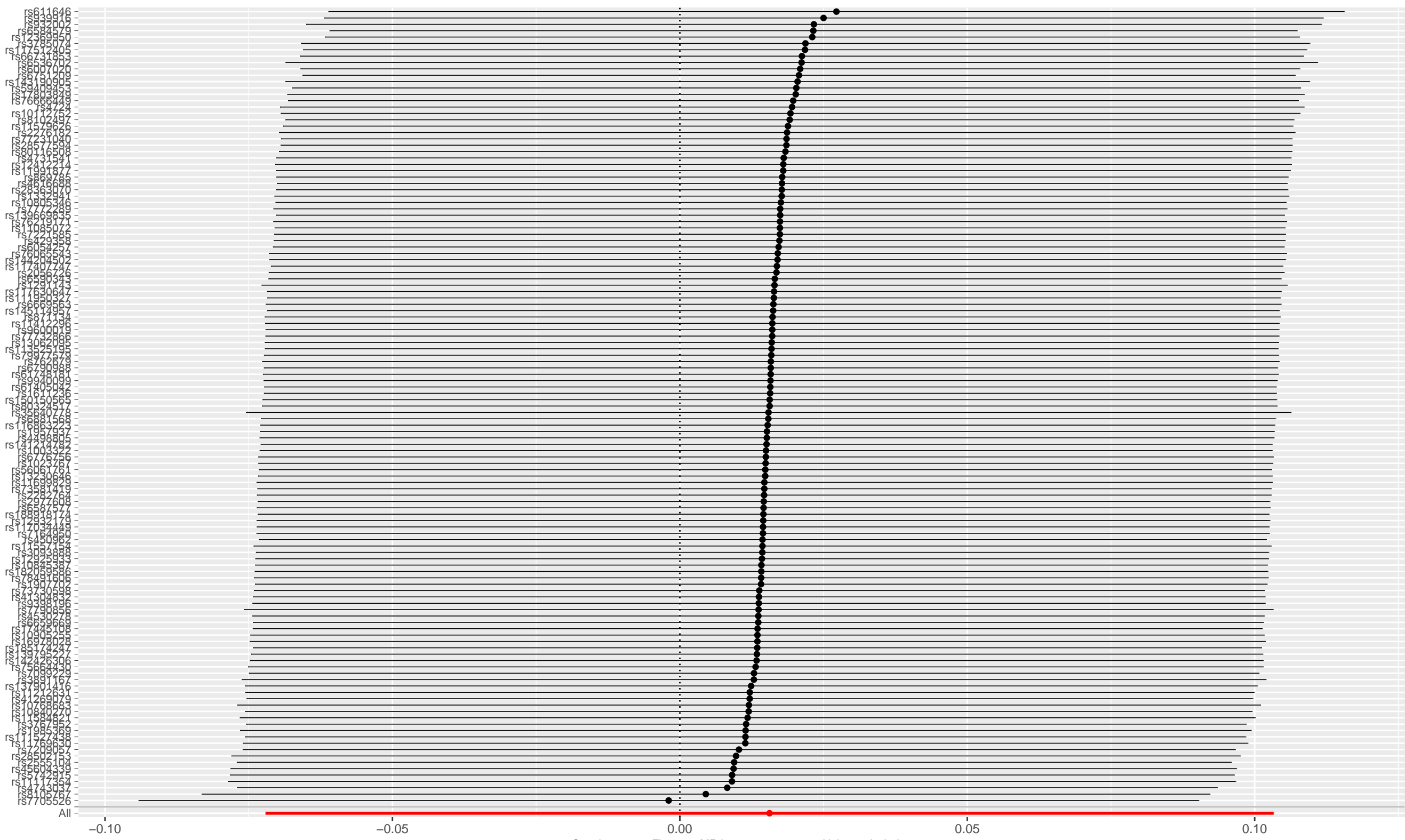

Supplement: Supplementary file 8 [file Data_Sheet_6.PDF]
